# Association between *Helicobacter pylori* Infection and Nasal Polyps: A Systematic Review and Meta-Analysis

**DOI:** 10.3390/microorganisms11061581

**Published:** 2023-06-14

**Authors:** Michael Doulberis, Jannis Kountouras, Thomas Stadler, Christian Meerwein, Stergios A. Polyzos, Hasan Kulaksiz, Michael H. Chapman, Gerhard Rogler, Daniele Riva, Ioannis Linas, John Kavaliotis, Evangelos Kazakos, Maria Mouratidou, Christos Liatsos, Apostolis Papaefthymiou

**Affiliations:** 1 Gastroklinik, Private Gastroenterological Practice, 8810 Horgen, Switzerland; doulberis@gmail.com (M.D.); kulaksiz@gastroklinik.ch (H.K.); 2 Department of Gastroenterology, University Hospital Zurich, 8091 Zurich, Switzerland; gerhard.rogler@usz.ch; 3 Division of Gastroenterology and Hepatology, Medical University Department, Kantonsspital Aarau, 5001 Aarau, Switzerland; 4 Department of Internal Medicine, Second Medical Clinic, Ippokration Hospital, Aristotle University of Thessaloniki, 54642 Thessaloniki, Macedonia, Greece; kavagrc@gmail.com (J.K.); ekazakos@gmail.com (E.K.); marysia.mouratidou@gmail.com (M.M.); 5 Department of Otorhinolaryngology, Head and Neck Surgery, University Hospital Zurich, 8091 Zurich, Switzerland; thomas.stadler@usz.ch (T.S.); christian.meerwein@usz.ch (C.M.); 6 First Laboratory of Pharmacology, School of Medicine, Aristotle University of Thessaloniki, 54124 Thessaloniki, Macedonia, Greece; spolyzos@auth.gr; 7 Pancreaticobiliary Medicine Unit, University College London Hospitals (UCLH), London NW1 2BU, UK; michael.chapman1@nhs.net (M.H.C.); appapaef@hotmail.com (A.P.); 8 Gastrocentro Plus, Private Gastroenterological Practice, 6900 Lugano, Switzerland; daniele.riva@hinmail.ch; 9 Gastroenterologische Gruppenpraxis, Private Gastroenterological Practice, 3011 Bern, Switzerland; ioannis.linas@hin.ch; 10 School of Healthcare Sciences, Midwifery Department, University of West Macedonia, 50100 Kozani, Macedonia, Greece; 11 Department of Gastroenterology, 401 General Military Hospital of Athens, 11525 Athens, Attica, Greece; cliatsos@yahoo.com

**Keywords:** *Helicobacter pylori*, nasal polyps, chronic rhinosinusitis, meta-analysis, association

## Abstract

Background: *Helicobacter pylori* (*H. pylori*) has definite or possible associations with multiple local and distant manifestations. *H. pylori* has been isolated from multiple sites throughout the body, including the nose. Clinical non-randomized studies with *H. pylori* report discrepant data regarding the association between *H. pylori* infection and nasal polyps. The aim of this first systematic review and meta-analysis was the assessment of the strength of the association between *H. pylori* infection and incidence of nasal polyps. Methods: We performed an electronic search in the three major medical databases, namely PubMed, EMBASE and Cochrane, to extract and analyze data as per PRISMA guidelines. Results: Out of 57 articles, 12 studies were graded as good quality for analysis. Male-to-female ratio was 2:1, and age ranged between 17–78 years. The cumulative pooled rate of *H. pylori* infection in the nasal polyp group was 32.3% (controls 17.8%). The comparison between the two groups revealed a more significant incidence of *H. pylori* infection among the nasal polyp group (OR 4.12), though with high heterogeneity I^2^ = 66%. Subgroup analysis demonstrated that in European studies, the prevalence of *H. pylori* infection among the nasal polyp group was significantly higher than in controls, yielding null heterogeneity. Subgroup analysis based on immunohistochemistry resulted in null heterogeneity with preserving a statistically significant difference in *H. pylori* infection prevalence between the groups. Conclusion: The present study revealed a positive association between *H. pylori* infection and nasal polyps.

## 1. Introduction

*Helicobacter pylori* (*H. pylori*) is one of the most common human bacterial infections worldwide with an estimated global prevalence of 58% [1,2], accounting for approximately 4.4 billion *H. pylori* infected individuals. It invariably causes histologic presence of gastritis [3] and is a recognized class I (definite) carcinogen [4]. Up to 3% of patients with clinical symptoms of *H. pylori* infection may develop gastric cancer [4], with over 1 million new cases of gastric cancer and nearly 800,000 deaths occurring in 2020, thereby making *H. pylori*-related gastric cancer the third leading cause of global cancer deaths [5]. Beyond the local well-established virulence of *H. pylori,* multiple extra-gastric manifestations have been attributed to *H. pylori* infection [3,6,7]. These manifestations include hematological, cardiovascular, respiratory, neurological, ophthalmic and metabolic syndrome-related disorders and other non-gastric neoplasms [3,8,9,10,11,12].

Interestingly, *H. pylori* has been identified in distant organs and tissues beyond the stomach, such as trabeculectomy specimens, colonic polyps and the nose [13,14,15].

The nasal and oral cavities have been described and regarded as primary or permanent reservoirs for the *H. pylori* infection, which may facilitate transmission to others [8,16]. *H. pylori* bacteria can access the nasal cavity by *H. pylori*-related oronasal, gastroesophageal [4,17,18,19] plus laryngopharyngeal reflux [20], thereby potentially contributing to the development of sinus and nasal disorders, including chronic rhinosinusitis and its related nasal polyps. Data indicate that patients with chronic rhinosinusitis may exhibit a high prevalence of gastroesophageal reflux, thus signifying a potential association between gastroesophageal reflux disease and chronic rhinosinusitis [21]. Likewise, *H. pylori* infection may be linked with chronic rhinosinusitis associated with nasal polyps [22]. The high prevalence (5–12%) of chronic inflammatory rhinosinusitis associated with nasal polyps in the general population globally is a substantial public health problem owing to the significant negative impact on the quality of life and the financial burden on patients, with limited effective treatment options [23,24]. Moreover, patients with nasal polyps usually remain undiagnosed and untreated in a timely manner, which may be due to an absence of disorder-related awareness. Thus, the potential impact of *H. pylori* infection on nasal polyp development and management may have significant clinical impact. However, the available scientific evidence regarding the association of *H. pylori* infection and nasal polyps yields contradictory results [16,22,25,26,27,28,29,30,31,32,33,34,35,36,37,38,39,40,41], and a systematic review with meta-analysis is lacking. The present study aimed to systematically collect and analyze good-quality existing clinical studies investigating the potential association between *H. pylori* infection and nasal polyps.

## 2. Materials and Methods

### 2.1. Protocol and Eligibility Criteria

The present systematic review and meta-analysis has been conducted according to the Preferred Reporting Items for Systematic Reviews and Meta-Analysis (PRISMA) 2020 guidelines (Supplementary Table S1) [42]. The protocol of this study has been submitted for registration at PROSPERO, the international prospective register of systematic reviews (Registration Number: CRD42023427528, University of York, UK) [43].

The selection of eligibility criteria was based on the well-established and validated PICO (population, intervention, control, and outcomes) model for systematic reviews [44]. Comparative, case-control studies were considered for the final analysis, once the below requirements were met: (A) patients: individuals with diagnosed intranasal polyps who underwent polyp resection and consented for sample investigation about *H. pylori* presence, using (B) polymerase chain reaction (PCR), immunohistochemistry (IHC) or rapid urease test (CLO) in the tissue sample of polyp/nasal mucosa; (C) comparators: patients with similar demographic characteristics and no present nasal polyps who were examined by otorhinolaryngologists for other indications and consented for *H. pylori* presence in nasal mucosa biopsies; (D) outcome: active *H. pylori* infection of the nasal mucosa based on a validated test. Studies not written in the English language, missing crucial data for analysis, case reports or series, single-arm cohorts as well as animal studies were excluded.

### 2.2. Information Sources and Search Strategy

M.D. and A.P. from the research group initiated an in-depth electronic search of the three major medical libraries, i.e., EMBASE, MEDLINE/PubMed, and Cochrane, from inception to 21 November 2022 by utilizing the following Boolean search terms, modified according to the demands of each database: “*Helicobacter pylori*” AND “*Nasal polyps*”. No publication date limitation was set and only articles published in English were considered. Eligible articles from abstract screening and their reference lists were thoroughly reviewed for further information. Further relevant articles were identified from the bibliography of the retrieved articles and by using the “similar article” function within MEDLINE. Grey literature results, such as unpublished works, oral or poster presentations, and abstracts, were also considered and reviewed in the screening process. Once relevant missing data were identified, e-mail(s) were sent to the first or the corresponding authors. A database of the obtained articles was kept and managed with the Reference Manager Endnote 20 for Mac (Clarivate Analytics). In cases of manifold publications from the same research group, only the most recent and complete (i.e., final results vs. interim analysis) article was considered.

### 2.3. Data Extraction and Quality Assessment

Data regarding study-, intervention- and participant-related parameters were collected into a standardized form by the first and last author (M.D. and A.P.), while a further author (S.P.) checked the two independent datasets for any inconsistency. Disagreements were settled after intervention from a senior investigator (J.K.) in order to obtain a consensus.

Moreover, quality assessment was conducted out independently by two authors (T.S. and A.P.) by utilizing the well-established and validated so-called Newcastle-Ottawa Scale (NOS) [45] (Supplementary Table S2). 

### 2.4. Outcomes

The primary outcome of the present meta-analysis was the prevalence of active *H. pylori* infection in nasal polyps’ arm compared to nasal polyps without *H. pylori* presence in controls.

### 2.5. Statistical Analysis and Data Synthesis

The study outcomes were pooled and compared between the two groups through a random-effects model based on the DerSimonian and Laird test [46], and results were expressed in terms of odds ratio (OR) and 95% confidence interval (CI), when appropriate.

The presence of heterogeneity was calculated through I^2^ tests, with I^2^ < 30% interpreted as low-level heterogeneity and I^2^ between 30% and 60% as moderate heterogeneity. Any potential publication bias was verified through the visual assessment of funnel plots.

Subgroup analysis based on the different study areas and the diagnostic method used to detect *H. pylori* infection *I* (PCR, immunohistochemistry, CLO test) was performed to assess for the source of heterogeneity.

All statistical analyses were conducted using the Review Manager “RevMan” version 5 from the Cochrane collaboration [47]. For all calculations, a two-tailed *p* value of less than 0.05 was considered statistically significant.

### 2.6. Quality of Evidence

The quality of the provided evidence was rated based on GRADE (Grading of Recommendations, Assessment, Development and Evaluations) criteria [48].

## 3. Results

### 3.1. Characteristics of Included Studies

**Figure 1:**
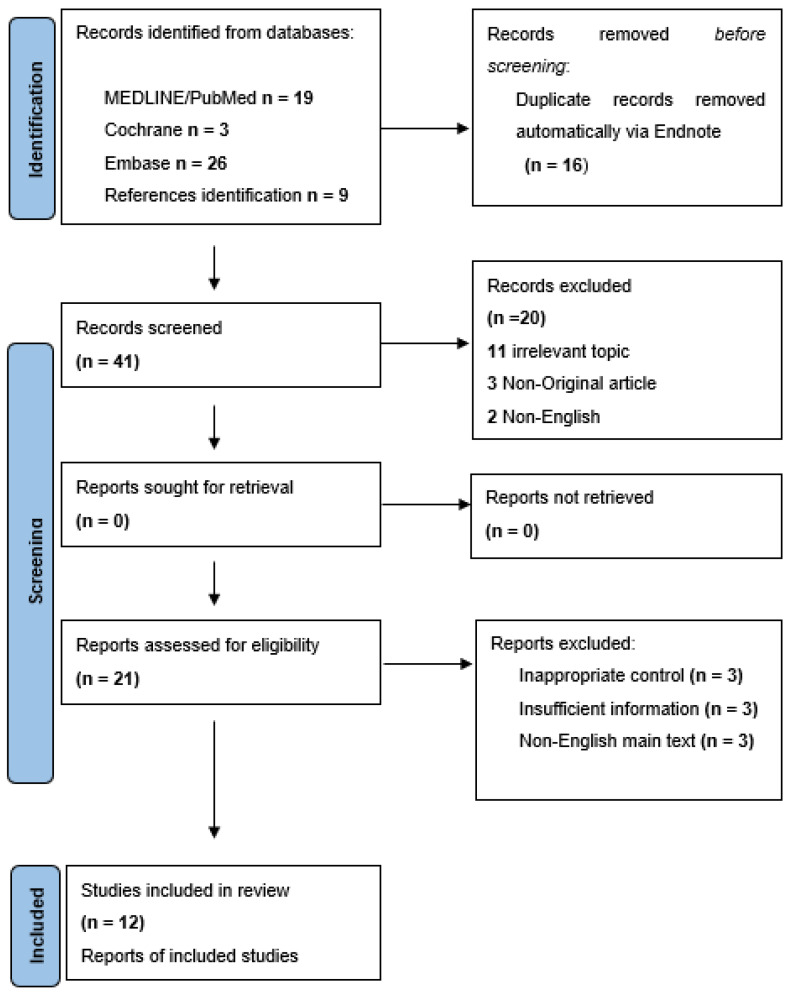
PRISMA Flowchart depicting the selection algorithm of the final eligible studies.

The initial research yielded 57 individual articles relative to the search terms. After considering the exclusion criteria, a total of 12 studies (710 patients) were eligible for analysis. Figure 1 depicts the PRISMA flowchart, and Table 1 summarizes the main characteristics of the included studies [16,22,25,26,27,28,29,31,33,34,35,49]. A total of 389 patients had nasal polyps, and 321 were included in the control group without polyps. All of the studies were prospective, with *H. pylori* investigation based on a predefined protocol. The majority of studies (seven studies, 430 patients) were conducted in Asian countries [16,26,27,28,29,31,34], four (208 patients) in Europe [22,25,33,49], whereas one Egyptian study (72 patients) represented Africa [35]. Five studies (284 patients) were performed in countries around the Mediterranean Sea.

The male-to-female ratio was 2:1, and the age ranged between 17 and 78 years. Six studies used tissue PCR to diagnose active *H. pylori* infection [25,26,27,29,35,49] whereas four used IHC. In two of them [29,34] (Table 1), the authors used the CLO test as a second technique to increase the rate of *H. pylori* infection diagnosis [28,34]. Finally, in five studies [25,26,28,31,32], the investigators assessed the existence of serum anti-*H. pylori* IgG antibodies using ELISA (enzyme-linked immunosorbent assay), and in one study they assessed *H. pylori* infection by stool antigen test [16].

### 3.2. Quality Assessment

All studies were graded to have good quality, scoring 6/8 according to NOS. The sole shortcoming for all studies was the absence of follow-up, though based on the studies’ design and defined outcomes, it is not expected to have changed the quality of the presented results (Supplementary Table S2).

### 3.3. Primary Outcome

**Figure 2:**
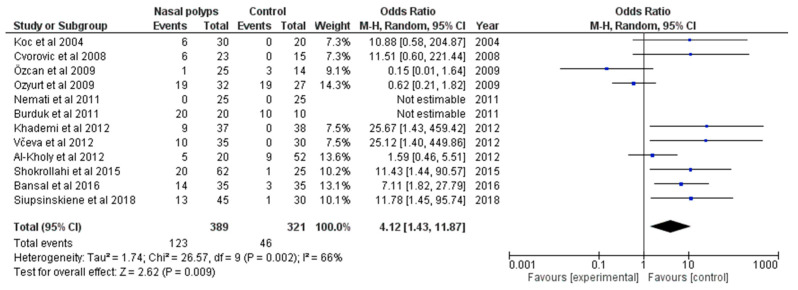
Forest plot of the included studies investigating the association between *H. pylori* infection and nasal polyps [16,22,25,26,27,28,29,31,33,35,49,50].

The cumulative pooled rate of *H. pylori* infection in the nasal polyp group was 32.3% (95% CI: 9.9–54.8) and in controls 17.8% (95% CI: 7.6–28.0). The comparison between two groups revealed a more significant incidence of *H. pylori* infection among the nasal polyp group with an OR of 4.12 (95 % CI: 1.43–11.87), though with high heterogeneity I^2^ = 66% (*p* = 0.002) (Figure 2).

### 3.4. Subgroup Analysis

**Figure 3:**
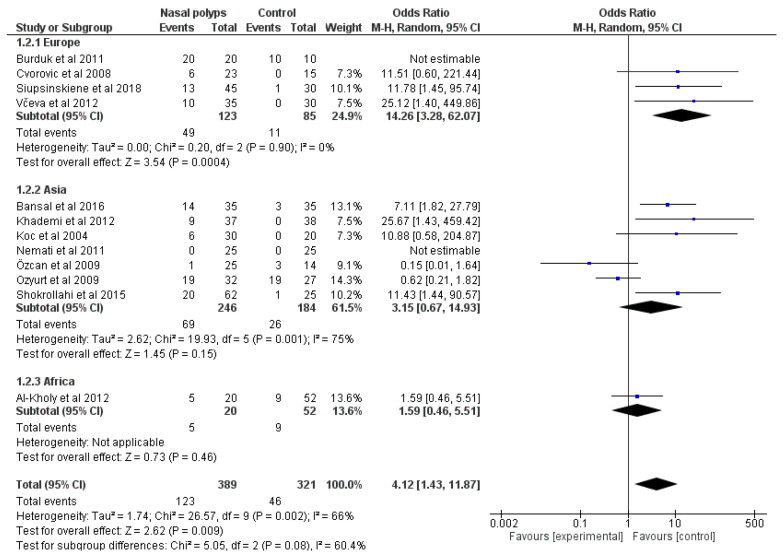
Forest plot of the subgroup analysis (based on continent of the included populations) reporting odd ratios of *H. pylori* infection among nasal polyp group and controls [16,22,25,27,28,29,31,33,35,49,50].

Two separate classifications were applied to perform subgroup analysis with regard to geographic origin, since prevalence of *H. pylori* infection exhibits also a geographic variation. The first continent-based categorization (Figure 3) indicated that in European studies, the prevalence of *H. pylori* infection among nasal polyp group was significantly higher than in controls, yielding null heterogeneity [OR: 14.26 (95% CI: 3.28–62.07; I^2^: 0%, *p* = 0.9)]. On the other hand, studies conducted in Asia did not show significant differences between subgroups and resulted in high heterogeneity [OR: 3.15 (95%CI: 0.67–14.93; I^2^: 75%, *p* = 0.001)]. The second classification included areas around the Mediterranean Sea (Supplementary Figure S1), where the prevalence of *H. pylori* infection between the nasal polyp group and controls did not differ with statistical significance [OR: 1.56 (95% CI: 0.38–6.52; I^2^:66%, *p* = 0.02)]. Studies from areas unrelated to the Mediterranean preserved the significant difference with optimal homogeneity [OR: 10.12 (95% CI: 4.11–24.89; I^2^: 0%, *p* = 0.95)].

Subgroup analysis based on the diagnostic technique (Supplementary Figure S2) resulted in null heterogeneity for IHC whilst preserving statistically significant difference in *H. pylori* infection prevalence between nasal polyp and control groups [OR: 9.06 (95% CI: 3.33–24.69; I^2^: 0%, *p* = 0.96)]. PCR and CLO in the tissue did not reduce heterogeneity and did not result in statistically significant outcomes [OR:7.75 (95%CI: 0.69–87.43; I^2^: 81%, *p* = 0.001) and OR: 1.57 (95% CI: 0.01–425.49; I^2^: 89%, *p* = 0.003), respectively].

### 3.5. Quality of Evidence

All studies were observational. Therefore, the quality of evidence was rated as low. No further causes for downgrading of the quality were identified. Thus, based on the meta-analysis, the low quality of evidence supported the comparisons among the presented modalities.

### 3.6. Publication Bias

The funnel plot considering the primary outcome is presented in Supplementary Figure S3, and the noticed symmetry indicates the absence of publication bias.

## 4. Discussion

To the best of our knowledge, this is the first systematic review and meta-analysis demonstrating that active *H. pylori* infection yields a statistically significant association with nasal polyps. In the subgroup analysis, European studies as well as studies with IHC as diagnostic modality of active *H. pylori* infection were characterized by absence of heterogeneity, without loss of the statistical significance. This positive association is in accordance with the findings of the majority of performed cohort studies, supporting a role of *H. pylori* in the pathophysiology of nasal polyps [16,22,25,26,31,33,39,49,50]. 

In our own study, the observed prevalence of *H. pylori* infection was 32.3%, which is within the reported range elsewhere [51], namely from 18.9% in Switzerland to 87.7% in Nigeria. However, this rate seems rather low, when the majority of the included studies are considered, which yields a considerably higher *H. pylori* infection prevalence: Africa’s estimated prevalence, 70.1%, and Western Asia’s, 66.6%.

In this respect, an interesting finding is the absence of association reported by two Turkish studies [27,28], a fact which is contradictory to the positive European studies. Since the prevalence of *H. pylori* infection is exceptionally high in the Turkish population, reaching 82.5% [52,53], this means that proving a difference in *H. pylori* prevalence among the groups in these studies [27,28] would require at least several hundred participants. Instead, these authors presented their results based on a small number of patients, and therefore the power of their studies was low. Further particularities or differences in the methodology used or ethnic/genetic background of the included patients may explain, at least partly, this variation. 

A further novel finding of our study was that IHC as a validated diagnostic modality revealed a statistical significance for active *H. pylori* infection with null heterogeneity. In this regard, histology has been repeatedly demonstrated as the practical diagnostic gold standard for detection of *H. pylori* [1,54]. IHC in particular yields an excellent sensitivity and specificity, reaching almost 100% [7]. Of special interest is the ability of IHC to detect the coccoid form of *H. pylori,* which forms for instance after proton pump inhibitor (PPI) treatment, where the conventional histology may be challenging in detecting the “disguised” microorganisms [55]. It is important to note that the *H. pylori* coccoid form is a critical factor for refractory *H. pylori* infection, the awareness of which helps to eradicate repeated treatment failure [56]. Once *H. pylori* is converted to its coccoid form, it enhances tolerance to the higher antibiotic concentrations, thereby supporting its survival [57,58]. In this regard, the policy of several studies for a combination of a second diagnostic technique of *H. pylori* infection can be endorsed, particularly when the aforementioned practical “gold standard” is not initially used or when the coccoid state of *H. pylori* is expected (for instance under PPI). 

Chronic rhinosinusitis with nasal polyps may be connected with *H. pylori* infection [22]. It appears to be one of the most prevalent chronic inflammatory conditions in adults aged < 45 years, affecting nearly 5 to 15% of the European population and 12 to 16% of the US population [59,60]. Moreover, chronic rhinosinusitis has a high economic impact on society in terms of direct and indirect costs [60].

Chronic rhinosinusitis phenotypes are classically divided into with or without nasal polyps mainly based on the presence of polyps, and both innate and adaptive immunity systems contribute to the heterogeneous pathogenesis of chronic rhinosinusitis [61,62,63]. According to our results, *H. pylori* infection seems to be connected with chronic rhinosinusitis phenotype associated with nasal polyps.

From a clinical setting, chronic rhinosinusitis involves the presence of two or more symptoms, including nasal obstruction and anterior or posterior nasal discharge for at least 12 weeks. Facial pain/pressure and dysosmia may be associated with these main complaints [64]. Cofactor contributing to the mucosal inflammation and the development of chronic rhinosinusitis includes gastroesophageal reflux [21]. In this respect, “migration” of *H. pylori* to the nose is hypothesized to be linked to gastroesophageal reflux [65]. This pathophysiological linkage has been also reported by Siupsinskiene et al. [22] in a high number of patients of both groups. However, such findings could not be evaluated within this meta-analysis due to the non-standard reporting in the included original articles.

Nasal polyps are categorized into three distinct groups: diffuse, localized and systemic. The local ones are either reactive-inflammatory or neoplastic. Although the etiology of nasal polyps remains unclear, infectious agents (including viruses, bacteria such as *H. pylori*, or fungi) may be potential primary factors activating nasal epithelial cells. Clinically, nasal obstruction, olfactory dysfunction including anosmia/hyposmia, rhinorrhea, postnasal drainage, headaches, facial pain or pressure, sleep disturbances and impaired quality of life comprise the main symptoms [66]. Typical findings of intranasal examination include mostly bilateral, mobile, smooth grey and semi-translucent polypoid masses that frequently originate in the ethmoid sinuses or the middle meatus. 

Differential diagnosis is important to rule out benign or malignant polyps. Certainly, nasal polyps ranging from benign lesions to malignant nasal tumors warrant such differential diagnosis, thereby justifying histological examination of all polyps [67]. Our meta-analysis was not able to evaluate the link with neoplastic polyps, which requires further study.

Regarding, the mechanism(s) potentially involved in the pathophysiology of nasal polyps, some data indicate that oxidative stress plays a substantial role in the pathophysiology of intranasal polyps. High levels of free radical-mediated lipid peroxidation metabolites have been found in nasal polyps and have been repeatedly linked to the severity of nasal polyps [68,69,70]. Specifically, nitric oxide is a free radical that is kept in balance by the antioxidant defense system, which includes superoxide dismutase (SOD), catalase, and glutathione peroxidase. Increased levels of nitric oxide and decreased SOD were found in patients with nasal polyps compared to controls, suggesting the presence of free radical damage in nasal polyps [71]. In contrast, several promising antioxidants may offer benefit for chronic rhinosinusitis with nasal polyps [70]. Likewise, *H. pylori* infection, by inducing oxidative stress, may also play a role in the pathogenesis of both local and systemic disorders [72,73], possibly including chronic rhinosinusitis-related nasal polyps. In contrast, certain antioxidants display antimicrobial activities, including against *H. pylori* [74]. Thus, such antioxidants may also offer benefit for *H. pylori*-related chronic rhinosinusitis with nasal polyps, and further research is needed to elucidate this consideration. 

Proinflammatory cytokines and growth factors play important roles in the persistence of mucosal inflammation associated with nasal polyps. Loss of the immune barrier, including dysfunction or impairment of the nasal epithelial barrier caused by oxidative stress [75] and decreased production of essential antimicrobial substances and responses, is a feature of several forms of chronic rhinosinusitis. One type of chronic rhinosinusitis with polyps observed worldwide is driven by the interleukin (IL)-5 and IL-13 coming from T helper (Th)2 lymphocytes, type 2 innate lymphoid cells and possibly mast cells. Novel biologic agents blocking the production or action of such cytokines, including, for instance, IL-5, IL-5Rα, IL-33 and immunoglobulin (Ig)E, have been developed and examined for treatment of chronic rhinosinusitis-related nasal polyps [76]. Of note, a variety of cytokines induced by *H. pylori* include, among others, IL-5 and IL-13 [77]. Likewise, IgE may play a pathogenetic role in both *H. pylori*-induced gastric pathologies and urticaria [78,79]. Of note, urticaria is linked with chronic rhinosinusitis with nasal polyps [80], and therefore *H. pylori* infection may be related with the presence and persistence of urticaria [81] and supports another indication for eradication [81,82,83,84].

In Western societies, nasal polyps are predominantly the consequence of a Th2 cell-driven eosinophilia, IgE inflammation and raised IL-5, often associated with environmental and/or seasonal allergic triggers. Further pathogenetic theories hypothesize a fungi-driven inflammatory process as well as an inflammatory response triggered by exotoxins through *Staphylococcus aureus* infections [85]. *H. pylori* is another microorganism known to induce an inflammatory cascade of cytokines [77] and mixed Th1/Th17 immune response and differentiation of Th2 cells [86].

Beyond *H. pylori*-related nasal polyps, *H. pylori* infection is also involved in the pathogenesis of colonic polyp development and progression. Our group showed [87,88] by means of histology (Cresyl violet and IHC) the presence of *H. pylori* bacteria in malignant colonic neoplasms (34/41 patients, 82.9%). By extending these preliminary data to the inclusion of a further 50 patients (28 men, mean age 71.3 ± 9.7 years) with colorectal carcinoma as well as 25 patients (13 men, mean age 72.8 ± 10.1 years) with colonic polyps, it was demonstrated that *H. pylori* was identified in malignant and polyp tissues of patients in 84% and 64%, respectively. A relevant recent systematic review and meta-analysis investigated the abovementioned association of *H. pylori* infection and colonic polyps [89]. The authors reported after the inclusion of 17 studies (*n* = 322,395) that *H. pylori* infection was independently associated with colorectal polyps, albeit with high heterogeneity (OR: 1.67, 95%, *p* < 0.001; I^2^ = 73%). Likewise, a similar association has been described for *H. pylori* infection and gall bladder polyps [90].

Despite the clear message of a positive association between *H. pylori* infection and nasal polyps, this first systematic review and meta-analysis has some limitations. All the included studies are case-control studies with high heterogeneity. The geographical coverage of eligible studies is almost exclusively from Eastern Europe/Mediterranean and Middle East, whereas studies from other continents such as Oceania, America and others are absent. Furthermore, the evidence presented cannot prove any direct causative association between *H. pylori* infection and nasal polyps.

## 5. Conclusions

This is the first systematic review investigating and robustly supporting the association between *H. pylori* infection and nasal polyps. Further basic research and mechanistic studies are warranted to investigate a direct causative link in order to consider screening and eradication protocols in the management of nasal polyps.

## Figures and Tables

**Table 1:** Main characteristics of included studies.

Study	Year	Country	Study Type	Study Period	Gender (Male, %)	Age of Cases (Mean ± SD/Median, Range)	Age of Controls (Mean ± SD/Median, Range)	Test for Gastric *H. pylori* Infection
[35]	2012	Εgypt	prospective case-control	2010–2012	173 (59.2%)	29.5 ± 15.4		PCR
[16]	2016	India	prospective case-control	2011–2012	52 (74.3%)	32 ± 11.0	28.2 ± 8.0	IHC
[33]	2008	Serbia	prospective case-control					IHC
[31]	2004	Turkey	prospective case-control	2001–2003	17 (56.7%)	41.5 (13–62)	39.5 (25–58)	IHC
[28]	2009	Turkey	prospective case-control		26 (66.6%)	37.56 (19–69)	31 (17–49)	CLO/IHC
[27]	2009	Τurkey	prospective case-control	2007–2008	28 (93.2%)	34.48 (17–77)	22.3 (19–28)	PCR
[26]	2015	Iran	prospective case-control	2007–2008	55 (63.2%)	37.5 ± 13.7	31 ± 11.5	PCR
[22]	2018	Lithuania	prospective case-control	2011–2012	29 (70.6%)	51.8 ± 14.9	41.6 ± 17.6	IHC
[25]	2012	Croatia	prospective case-control		25 (71.4%)	54 (27–78)	42.5 (19–75)	PCR
[34]	2012	Iran	prospective case-control	2006–2008	52 (69.3%)	41.27 (16–76)	24.4 (17–59)	PCR/CLO
[49]	2011	Poland	prospective case-control		19 (63.3%)	48. 7 (23–71)	43.3 (19–62)	PCR
[29]	2011	Iran	prospective case-control	2010		32.12 ± 14.08	24.36 ± 5.91	PCR/CLO

CLO, urease rapid test; *Hp*-I, *Helicobacter pylori* infection; IHC, immunohistochemistry; PCR, polymerase chain reaction; SD, standard deviation.

## Data Availability

The data presented in this study are available on request from the corresponding author.

## Supplementary Materials

The following supporting information can be downloaded at https://www.mdpi.com/article/10.3390/microorganisms11061581/s1, Figure S1: The second classification included areas around the Mediterranean Sea. Figure S2: Subgroup analysis based on the diagnostic technique. Figure S3: The funnel plot of publication bias considering the primary outcome. Table S1: PRISMA checklist. Table S2: Quality assessment by Newcastle-Ottawa Scale (NOS).

## Abbreviations

CI, confidence interval; CLO, rapid urease test; ELISA, enzyme-linked immunosorbent assay; GC, gastric cancer; GRADE, grading of recommendations, assessment, development and evaluations; *H. pylori, Helicobacter pylori*; *H. pylori* infection, *Helicobacter pylori* infection; IHC, immunohistochemistry; IG, immunoglobulin; IL, interleukin; NOS, Newcastle-Ottawa scale; OR, odds ratio; PCR, polymerase chain reaction; PICO, population, intervention, control, and outcomes; PRISMA, preferred reporting items for systematic reviews and meta-analysis; Th, T helper lymphocytes.
