# Supplementary material for: Association between Helicobacter pylori Infection and Nasal Polyps: A Systematic Review and Meta-Analysis
Source: Microorganisms. 2023 Jun 14;11(6):1581. doi: 10.3390/microorganisms11061581 (PMC10301196; doi:10.3390/microorganisms11061581)
Supplement: Supplementary file 1 [file microorganisms-11-01581-s001.zip › Suppl Table 2.pdf]

Supplementary Table 2. Risk of bias assessment using the Newcastle-Ottawa scale

STUDY	Representativeness of the exposed cohort	Non-exposed cohort	Ascertainment of exposure	Demonstration that outcome of interest was not present at start of study	Comparability of cases and controls on the basis of the design or analysis	Assessment of outcome	Was follow-up long enough for outcomes to occur	Adequacy of follow up	Total score
Al-Kholy et al	*	*	*	*	*	*	0	0	6/8
Bansal et al	*	*	*	*	*	*	0	0	6/8
Cvorovic et al	*	*	*	*	*	*	0	0	6/8
Khademi et al	*	*	*	*	*	*	0	0	6/8
Koc et al	*	*	*	*	*	*	0	0	6/8
Özcan et al	*	*	*	*	*	*	0	0	6/8
Ozyurt et al	*	*	*	*	*	*	0	0	6/8
Shokrollahi et al	*	*	*	*	*	*	0	0	6/8
Siupsinskiene et al	*	*	*	*	*	*	0	0	6/8
Včeva et al	*	*	*	*	*	*	0	0	6/8
Burduk et al	*	*	*	*	*	*	0	0	6/8
Nemati et al	*	*	*	*	*	*	0	0	6/8
