# Supplementary figures and images for: Association between Helicobacter pylori Infection and Nasal Polyps: A Systematic Review and Meta-Analysis
Source: Microorganisms. 2023 Jun 14;11(6):1581. doi: 10.3390/microorganisms11061581 (PMC10301196; doi:10.3390/microorganisms11061581)

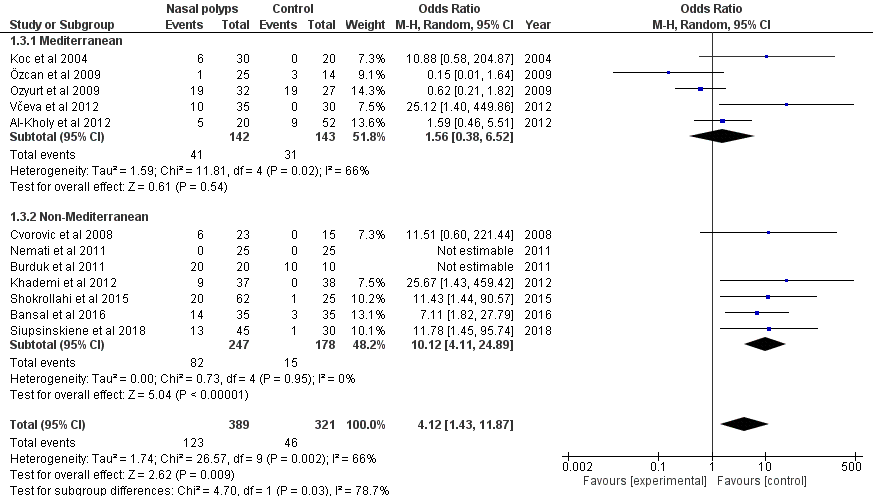

Supplement: Supplementary file 1 [file microorganisms-11-01581-s001.zip › Supl Figure S1.png]

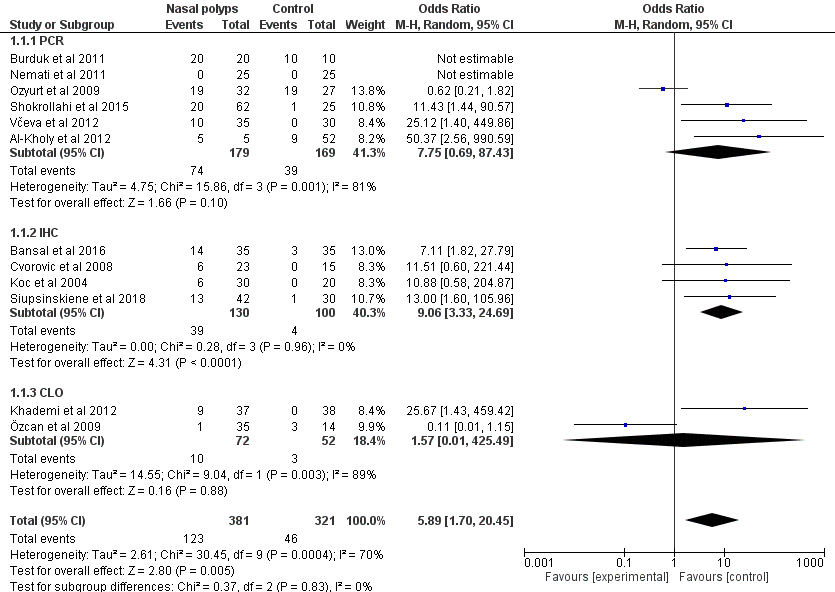

Supplement: Supplementary file 1 [file microorganisms-11-01581-s001.zip › Supl Figure S2.png]

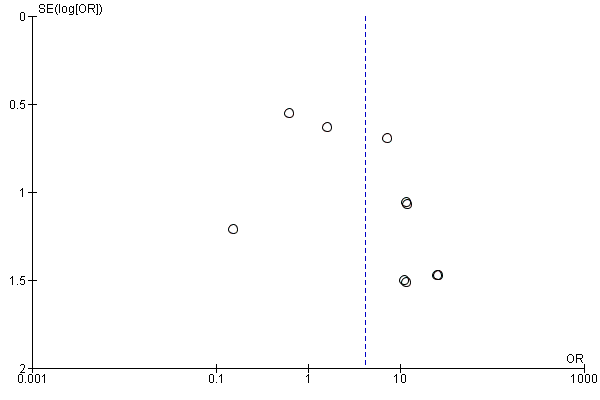

Supplement: Supplementary file 1 [file microorganisms-11-01581-s001.zip › Suppl Figure S3.png]
